# Sanitary Laws Enacted by the Twenty-Eighth Legislature

**Published:** 1903-04

**Authors:** 


### SANITARY LAWS ENACTED BY THE TWENTY-EIGHTH LEGISLATURE.

THE VITAL STATISTICS BILL as it passed, emasculated in Senate Committee:

S. S. B. No. 168.] [By Harper.

A BILL

TO BE ENTITLED.

An Act to carry into effect Section 32 of Article 16, of the Constitution of the State of Texas, in relation to a State Board of Health and Vital Statistics; to change the name of the Quarantine Department to the Department of Public Health and Vital Statistics, and to create and establish a State Bureau of Vital Statistics within said department, and to provide for the record and preservation of its vital statistics, etc.

Section 1. Be it enacted by the Legislature of the State of Texas: That a Bureau of Vital Statistics is hereby created and established within the Quarantine Department, and that the name of said department is hereby changed to the Department of Public Health and Vital Statistics.

Sec. 2. All physicians, surgeons or accoucheurs who may attend at the birth of a child, or in the absence of such attendance, either parent of the child, shall report the fact to the clerk of the county court, together with the race to which the child belongs, its sex, and the name of the parents, whether foreign or native, whether still-born or alive, within ten days after said birth occurs, under a penalty of five dollars for each failure to do so, to be collected as other fines for misdemeanors are.

All physicians, surgeons, accoucheurs and coroners cognizant of a death, shall report the same, together with the race, nativity, sex, age, residence, whether alien or citizen, and the cause of death, to the clerk of the county court within ten days after the occurrence, under a penalty of not less than five dollars nor more than fifty dollars for each failure to do so; these data to be recorded as a part of the vital statistics of the county and State, and the clerk of the county court shall be paid by the county twenty-five cents for each birth or death so recorded, and he shall report monthly all these data to the Department of Public Health and Vital Statistics. In default of so reporting he shall be fined not less than fifty dollars for each offense.

